# Clinical performance of the urine‐based *TERT* promoter AbsoluteQ Digital PCR for non‐invasive detection of bladder cancer

**DOI:** 10.1002/1878-0261.70262

**Published:** 2026-05-05

**Authors:** Anna Nykel, Izabela Kubiak, Lena Rutkowska, Żaneta Kasprzyk, Łukasz Kępczyński, Michał Bednarek, Dariusz Sobieraj, Jacek Wilkosz, Piotr Kania, Adam Jędrzejczyk, Bogdan Kałużewski, Agnieszka Gach, Tadeusz Kałużewski

**Affiliations:** ^1^ Department of Genetics Polish Mother's Memorial Hospital Research Institute Lodz Poland; ^2^ Laboratory of Medical Genetics, R&D Division GENOS Sp. z o.o. Lodz Poland; ^3^ Department of Urology and Urological Oncology John Paul II Podkarpackie Province Hospital in Krosno Poland; ^4^ Second Department of Urology Medical University of Lodz Poland; ^5^ Department of Urology and Urologic Oncology Saint John Paul II Mazovian Regional Hospital in Siedlce Poland; ^6^ Department of Urology District Hospital Radomsko Poland

**Keywords:** bladder cancer, digital PCR, non‐invasive diagnostics, TERT promoter variants

## Abstract

Bladder cancer (BC) is the ninth most common cancer worldwide, with urothelial carcinoma accounting for approximately 90% of all cases and presenting predominantly as non‐muscle‐invasive disease. Due to its high recurrence rate and the need for long‐term surveillance, BC is associated with the highest lifetime treatment costs per patient among all cancers, making its effective management a significant clinical and economic challenge. The most frequently identified variants in the *TERT* gene promoter are c.‐124C>T (C228T) and c.‐146C>T (C250T), located within a region characterized by high guanine–cytosine (GC) content, which makes amplification challenging. We aimed to validate the AbsoluteQ Digital PCR assay for the detection of urine‐based *TERT* promoter variants for the diagnosis of urothelial bladder cancer and to assess its diagnostic performance in comparison with standard methods. Urine samples were collected from patients with histopathologically confirmed bladder cancer (*n* = 58) and compared with a control group (*n* = 55). The C228T and C250T variants were tested using the AbsoluteQ Digital PCR assay. Sensitivity, specificity, and predictive values were calculated to evaluate the performance of the assessed method. The AbsoluteQ Digital PCR demonstrated superior diagnostic performance compared to conventional Sanger sequencing for detecting *TERT* promoter variants, achieving a sensitivity of 89.65% (95% CI: 78.16–95.72) and a specificity of 100% (95% CI: 91.87–100), with no false positives observed. Given its robustness and clinical relevance, AbsoluteQ Digital PCR is emerging as a promising tool for non‐invasive molecular diagnostics targeting *TERT* promoter variants.

## Graphical abstract

A urine‐based digital PCR assay targeting two hotspot *TERT* promoter variants detected bladder cancer with high sensitivity and no false positives in this case–control cohort. The streamlined AbsoluteQ workflow outperformed Sanger sequencing and supports non‐invasive molecular testing for bladder cancer detection.

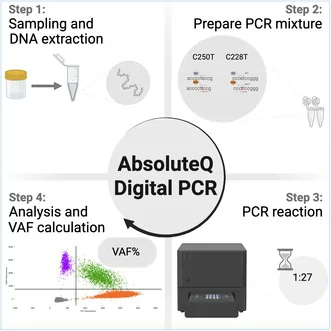

**Abbreviations**
BC: bladder cancer
CI: confidence interval
CIS: carcinoma *in situ*
ddPCR: droplet digital PCR
DNA: deoxyribonucleic acid
dPCR: digital PCR
FFPE: formalin‐fixed paraffin‐embedded
GC: guanine‐cytosine
LOD: limit of detection
MIBC: muscle‐invasive bladder cancer
NGS: next‐generation sequencing
NHGUC: negative for high‐grade urothelial carcinoma
NMIBC: non‐muscle‐invasive bladder cancer
PCR: polymerase chain reaction
RMSE: root mean square error
SD: standard deviation
TERT: telomerase reverse transcriptase
VAF: variant allele frequency

## 1 Introduction

### 1.1 Bladder cancer epidemiology

Bladder cancer (BC) is the ninth most common cancer worldwide, with an estimated 613 791 new cases and 220 349 deaths in 2022 worldwide [1]. In 2021, 7043 new cases of BC were recorded in Poland (5301 in men—ranking 5th, and 1742 in women—ranking 14th in malignancy incidence in Poland), out of whom 3867 died (2978 men and 889 women) [2]. Urothelial carcinoma is the predominant histological form of bladder cancer, representing 90% of all cases. It is classified into two main categories: non‐muscle‐invasive (NMIBC), which accounts for around two‐thirds of cases, and muscle‐invasive (MIBC) [3]. The management of urothelial cancer, with all its complexities, has recently gained significant attention, partly due to the fact that BC incurs the highest lifetime treatment costs per patient among all cancer types [4].

### 1.2 The current state of diagnostics

The current gold standard for diagnosing BC is cystoscopic visualization of bladder tumors, which has an overall sensitivity of 62%–84% and specificity of 43%–98% [5]. However, cystoscopy has a limited ability to diagnose small papillary BC and BC carcinoma *in situ* (CIS) and is cost‐intensive, invasive, and operator‐dependent [6]. Among non‐invasive tests, urine cytology is the most reliable among urinary biomarker tools for detecting exfoliated tumor cells in the urine, with an overall sensitivity and specificity of up to 48% and 86%, respectively [7, 8]. It complements both primary diagnosis and recurrence surveillance of high‐grade or CIS BC treated with intravesical therapy, with 38%–90% sensitivity and 98%–100% specificity. However, its sensitivity for low‐grade BC is only 4%–31%, and the 12% false‐positive rate reflects a poor ability to screen out patients with inflammation, atypical urothelial cells, or a history of other cancer therapies [7, 8, 9, 10]. Hence, there is a need to identify biomarkers that can improve the diagnosis and risk stratification of BC. Several urinary biomarkers have been evaluated for BC screening in high‐risk populations, but none has shown sufficient diagnostic accuracy and cost‐effectiveness to justify implementation in routine practice [11, 12, 13]. Accordingly, major clinical guidelines do not recommend routine screening for bladder cancer (EAU Guidelines on Non‐muscle‐invasive Bladder Cancer (TaT1 and CIS) 2025). However, several biomarkers achieved high clinical utility in disease monitoring including XPERT BC® MONITOR (sensitivity 52%–91% overall and 79%–100% for high‐grade recurrences; specificity 41%–91% overall) [14], EpiCheck™ (sensitivity 62%–90% overall and 95% for high‐grade; specificity 82%–88%) [15], CX BLADDER (sensitivity 93% overall and 95% for high‐grade; specificity 61%) [16] as well as UROMONITOR (sensitivity 49%–93% and specificity 86%–99%) [17] and Galeas Bladder (sensitivity 86% overall, 100% for high‐grade and specificity 63%) [18] of which *TERT* gene is one of the major targets.

### 1.3 Role of *TERT* variants in bladder cancer

The *TERT* gene encodes the catalytic subunit of the telomerase enzyme, which exhibits reverse transcriptase activity. Telomerase plays a fundamental role in maintaining telomere length and preserving chromosomal stability. Telomeres consist of tandem DNA repeat sequences located at the distal ends of chromosomes protected by specialized protein complexes that prevent their degradation and maintain genomic integrity during DNA replication. With each cell division, telomeres progressively shorten, ultimately bringing the cell closer to a critical threshold at which coding regions of the genome may become vulnerable to damage, leading to genomic instability, cellular senescence, or apoptosis. Telomerase activity represents the primary mechanism that counteracts this process. In humans, telomerase is expressed in embryonic stem cells and is subsequently downregulated in somatic cells during differentiation. Increased expression of the *TERT* gene restores telomerase activity, enabling telomere elongation and repair. Telomerase activity, driven by *TERT*, is detected in over 90% of human cancers and is considered a key factor in the acquisition of cellular immortality [19, 20, 21, 22, 23]. Variants in the *TERT* gene promoter are the most common genetic abnormalities detected in DNA samples isolated from tumor tissue in cases of bladder cancer occurring regardless of histopathological type and clinical status [24, 25]. The most frequently identified variants in the *TERT* gene promoter are c.‐124C>T (C228T) and c.‐146C>T (C250T), both of which are located upstream of the transcription start site and have been implicated in telomerase reactivation across multiple cancer types [26]. The promoter region of the *TERT* gene is characterized by a high guanine–cytosine (GC) content (> 80%), which facilitates the formation of stable secondary DNA structures, such as hairpins [27]. This feature has significant structural and functional implications, particularly in the context of somatic variants such as C228T and C250T. In practical terms, the high GC content of the *TERT* promoter presents a major challenge for molecular assays.

### 1.4 Digital PCR technology

Digital PCR (dPCR) offers superior sensitivity and precision for detecting and quantifying low‐abundance DNA sequences, including hotspot variants in the promoter region of the *TERT* gene [28]. In contrast to conventional PCR methods, dPCR partitions the DNA sample into thousands of reactions in microchambers, enabling absolute quantification of target sequences without the need for standard curves [29]. This makes digital PCR particularly suitable for identifying low‐frequency somatic variants, such as C228T and C250T. These variants occur within a highly GC‐rich region of the *TERT* promoter, which can pose challenges for amplification using traditional PCR techniques [30]. However, dPCR can overcome these limitations through increased reaction efficiency and the use of optimized reagents capable of resolving GC‐rich templates. Digital PCR technology enables precise measurement of variant allele frequencies (VAF), enabling sensitive detection of variants in heterogeneous samples, such as urinary sediments.

### 1.5 Study goal

This study aimed to validate the AbsoluteQ Digital PCR for the detection of *TERT* promoter variants in urinary sediment samples for the diagnosis of urothelial bladder cancer and to evaluate its diagnostic performance in comparison with standard methods. This approach is expected to enhance non‐invasive diagnostic strategies and improve early detection of bladder cancer through straightforward molecular analysis of urine samples.

## 2 Materials and methods

### 2.1 Patient characteristics and study design

This was an observational case–control study. In total, 113 participants were included: 58 cases and 55 controls. Cases comprised urine specimens submitted by referring urologists from patients with newly diagnosed, histopathologically confirmed bladder cancer, regardless of tumor stage or grade. The samples were collected from April 2020 to March 2023 at the Polish Mother's Memorial Hospital Research Institute. Eligibility required the ability to obtain a urine sample shortly after diagnosis. Patients were excluded if they had a prior urothelial malignancy in remission or had previously undergone cystectomy, which precluded urine collection from a native bladder. Controls were recruited as volunteer participants among individuals attending a urine sediment cytology assessment for various indications. Controls eligible for inclusion had no known history of bladder cancer and a urine cytology result reported as NHGUC (Negative for High‐Grade Urothelial Carcinoma). All participants provided urine sediment samples in sterile containers. An overview of patient characteristics is presented in Table 1.

**Table 1:** Summary of baseline characteristics of the study population.

	Case group	Control group	Total study cohort	*P*
Patients	58 (51.33%)	55 (48.67%)	113 (100%)	‐
Male	54 (47.78%)	2 (1.77%)	56 (49.55%)	< 0.0001
Female	4 (3.55%)	53 (46.90%)	57 (50.45%)	
Mean age	72 years	53 years	59 years	‐
Male	72 years	63 years	72 years	< 0.0001
Female	67 years	52 years	53 years	
Bladder cancer	58 (100.00%)	‐	58 (100.00%)	‐
Low‐grade	36 (62.07%)	‐	36 (62.07%)	‐
High‐grade	22 (37.93%)	‐	22 (37.93%)	‐

*Note: P*‐values were calculated using Fisher's exact test for sex and Welch's *t*‐test for age.

### 2.2 Ethics approval and consent to participate

The study protocol was confirmed by the Polish Mother's Memorial Hospital Research Institute's Bioethics Committee (approval number 62/2024). All the procedures performed in this study followed the principles of the Declaration of Helsinki. All participants provided written informed consent to participate in the study and agreed to the anonymous publication of the results.

### 2.3 Processing of urine samples and DNA extraction

Urine samples with a volume of 50–200 mL were collected in sterile, single‐use containers and transported to the laboratory under controlled temperature conditions. Samples were stored at 2°C–8 °C for up to 5 days prior to extraction to minimize degradation. DNA was extracted from urine sediment using a commercially available QIAamp DNA Mini Kit (QIAGEN Inc, Hilden, Germany), according to the manufacturer's instructions. The purity and concentration of extracted DNA samples were assessed using an Eppendorf BioSpectrometer basic (Eppendorf, Hamburg, Germany). DNA concentration ranged from 1.75 to 1072.7 ng/μl (median 39.7 ng/μl).

### 2.4 AbsoluteQ Digital PCR

To identify *TERT* common variants, Taqman SNP Assays Hs000000092_rm for C228T and Hs000000093_rm for C250T variant were used (Life Technologies, Carlsbad, CA). For each dPCR assay, FAM and VIC reporter dyes are designed (FAM for mutated and VIC for the wild‐type alleles). Digital PCR was performed using the AbsoluteQ Digital PCR System (Life Technologies, Carlsbad, CA). The DNA samples were measured using Nanodrop (Life Technologies, Carlsbad, CA) and diluted to 10 ng/μl. Reactions were prepared in 10 μL with 8 μL Quantstudio 3D Digital PCR Master Mix v2 (Life Technologies, Carlsbad, CA), 0.4 μL of 40× Taqman SNP Assays and 1–10 ng DNA template diluted in 1.6 μL. Clinical sample DNA was diluted, whenever possible, to a concentration of 10 ng/μl. For a subset of samples where this concentration could not be achieved, the DNA input was lower but remained no less than 1 ng per reaction. Each MAP plate well was loaded with 9 μL of PCR mix then overlaid with 15 μL Isolation Buffer. Gasket caps were placed and the plate was put into the AbsoluteQ Digital PCR and run with the following conditions: preheating at 96 °C for 10 min and 39 cycles consisting of denaturing at 96 °C for 5 s, followed by annealing/extension at 55 °C for 15 s. MAP partitions, thermal cycling, imaging and data analysis were performed with AbsoluteQ Control Software (v6.3.2). Samples were considered positive if at least three FAM fluorescence signals were detected. The threshold was set individually and manually for each reaction. The analytical sensitivity of the AbsoluteQ dPCR assays was determined using a series of DNA mixtures containing 0.1%, 1%, 10%, and 30% of either the C228T or C250T variant in wild‐type DNA. These mixtures were generated by combining clinical samples identified as wild type and heterozygous for the respective variant. Prior to mixing, all DNA samples were normalized to a concentration of 10 ng/μl to ensure uniform input across all dilution levels. Based on the absolute quantification of mutant and wild‐type DNA molecules obtained by AbsoluteQ digital PCR, variant allele frequency was calculated as the ratio of mutant allele copies/μl to the total number of allele copies/μl (mutant plus wild‐type), expressed as a percentage. Absolute copy numbers for each allele were derived directly from partitioned reactions without the need for external calibration curves, in accordance with the principles of digital PCR.

### 2.5 Sanger sequencing

The PCR was performed using PCR Master Mix (Promega, Madison, Wisconsin, USA), and primers designed as follows: forward 5′‐ CAGTGGATTCGCGGGCAC‐3′ and reverse 5′‐ CTGCCTGAAACTCGCGCC‐3′ (231 bp product size). The reaction was performed using 10–50 ng of DNA, under the following steps: 95°C for 5 min, 27 cycles of a three‐step program of 95 °C for 30 s, 62 °C for 45 s and 72 °C for 30 s and final amplification of 72 °C for 7 min. After PCR amplification products were cleaned and sequenced using BigDye Terminator v3.1 Cycle Sequencing Kit (Life Technologies, Carlsbad, CA) on a 3500 Genetic Analyzer instrument (Life Technologies, Carlsbad, CA). Data were analyzed using Mutation Surveyor Software v5.0.1. After obtaining digital PCR results, all 113 samples were subsequently validated by Sanger sequencing, which was performed on both forward and reverse strands. A result was considered positive when the variant was detected in both forward and reverse Sanger reads (reported in the Results section as “Sanger F + R”); an additional analysis was also conducted using a permissive definition in which positivity required detection in at least one of the two directions (reported as “Sanger F v. R“).

### 2.6 Statistical analysis

All statistical analyses were performed using Python version 3 and appropriate libraries for data manipulation and analysis (e.g., NumPy, pandas, sklearn, and matplotlib). Linear regression analysis was performed to evaluate the relationship between dilution percentages and variant allele frequency (VAF) values. Coefficient of determination (*R* ^2^) was calculated to assess the goodness of fit of the linear model. Additionally, the Root Mean Square Error (RMSE) was computed.

## 3 Results

### 3.1 Optimization of AbsoluteQ digital PCR assay

**Fig. 1:**
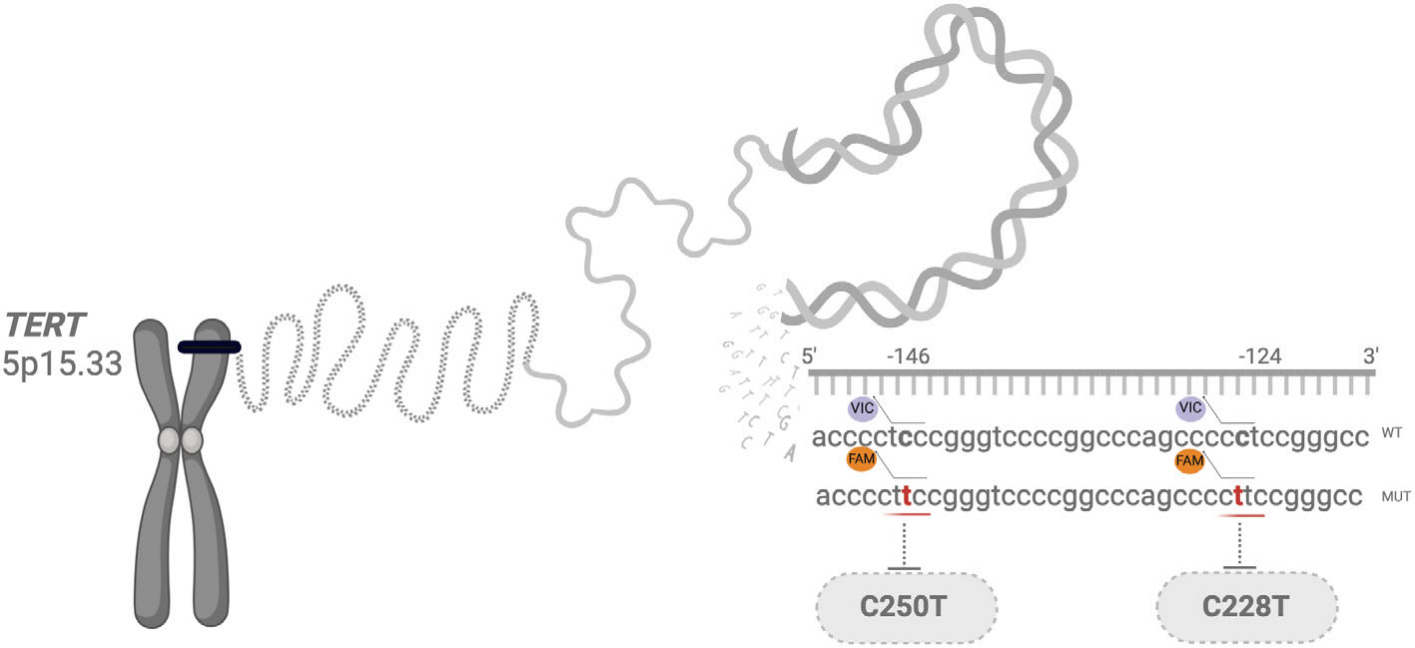
Schematic representation of the GC‐rich *TERT* promoter region highlighting hotspot variants C228T and C250T. MUT, mutated sequence; WT, wild type.

C228T and C250T variants are the most prevalent variants in the *TERT* promoter region, located 124 and 146 base pairs upstream of the transcription start site (Fig. 1). The promoter region of the *TERT* gene is highly GC‐rich, with a high guanine‐cytosine (GC) content exceeding 80% and easily forms a secondary structure, such as a hairpin structure, resulting in a poor amplification.

A series of reaction optimizations was performed for two assays: Hs000000092_rm for the C228T and Hs000000093_rm for the C250T variant. The first step involved testing the manufacturer‐recommended conditions for the AbsoluteQ Digital PCR System. The PCR mixture contained the AbsoluteQ Universal DNA Master Mix (Life Technologies, Carlsbad, CA), which is specifically designed for this platform. However, no amplification was observed under these conditions. Adjusting the assay concentrations and modifying the annealing temperature had no impact on the results (data not shown). The absence of an amplification product, regardless of reaction conditions, likely indicates the presence of inhibitors affecting reactions using the AbsoluteQ Digital PCR–dedicated Universal DNA Master Mix. As a second step, the Master Mix was replaced with QuantStudio 3D Digital PCR Master Mix v2 (Life Technologies, Carlsbad, CA), dedicated to the QuantStudio 3D Digital PCR System. Specific amplification was achieved for both assays using extended (54‐cycle) and shortened (39‐cycle) PCR protocols. A short amplification program consisting of 39 cycles was selected for its relative simplicity and robust performance. The final optimized conditions consisted of a preheating step at 96 °C for 10 min, followed by 39 cycles of denaturation at 96 °C for 5 s and annealing/extension at 55 °C for 15 s.

### 3.2 Limit of detection (LOD) assessment

**Fig. 2:**
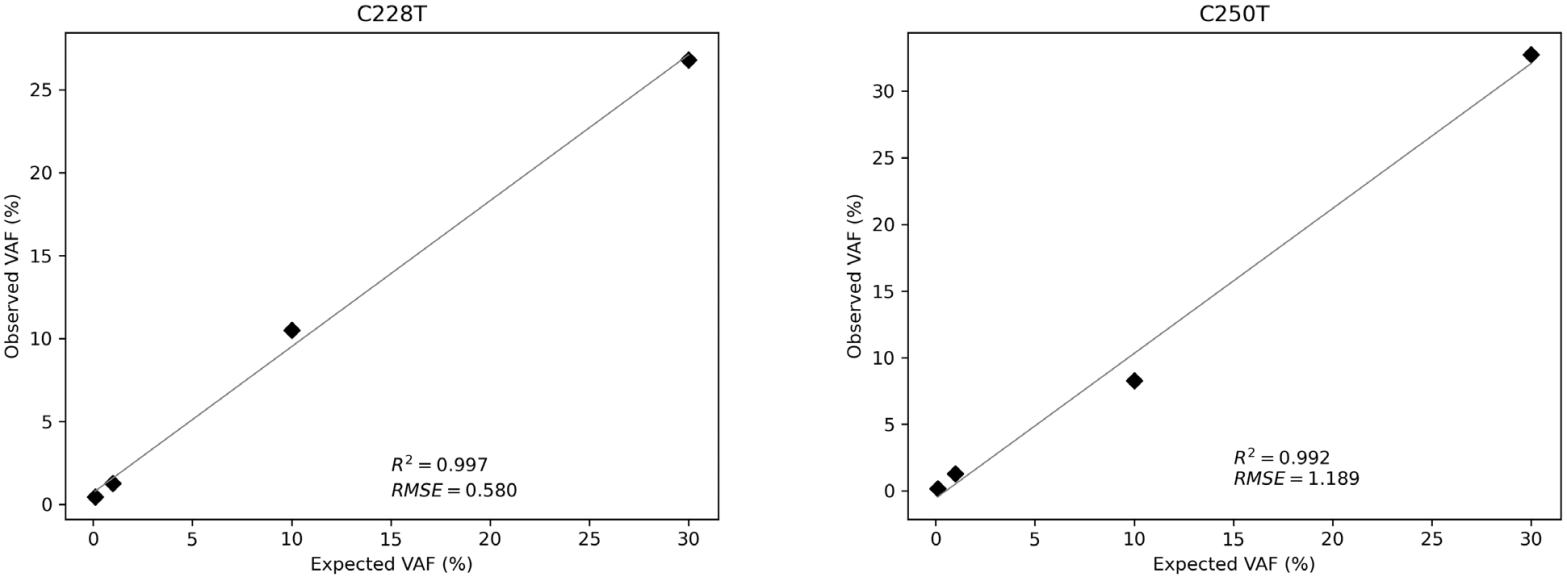
Correlation between expected and observed variant allele frequency (VAF) values for the C228T and C250T variants. Expected VAFs were generated by serial dilution of variant‐positive DNA into wild‐type DNA at 0.1%, 1%, 10%, and 30%. Each dilution was analyzed in triplicate (*n* = 3 technical replicates per dilution). Diamonds represent the mean observed VAF values. Solid lines indicate the linear regression fit used for quantitative assessment; the coefficient of determination (*R* ^2^) and root mean square error (RMSE) are shown in the panels. No formal significance test was applied.

**Fig. 3:**
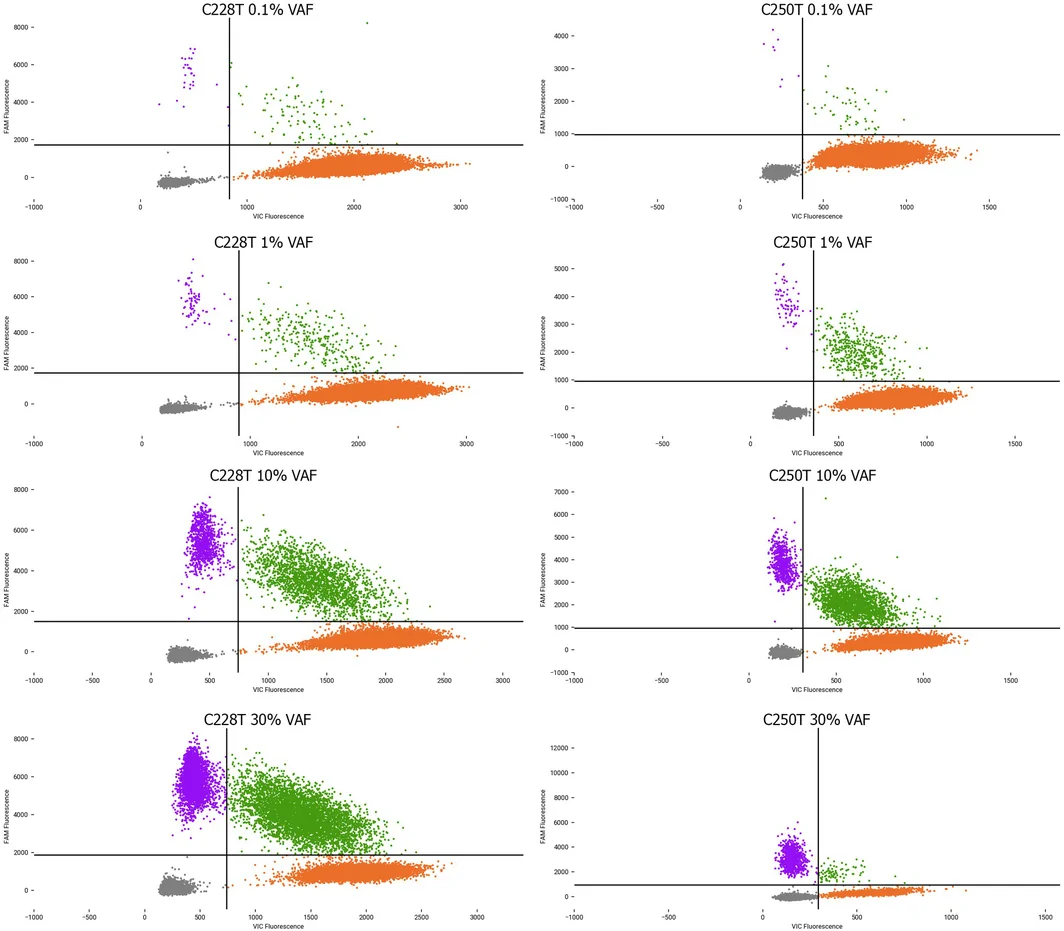
Representative scatter plots generated by digital PCR (dPCR) assays targeting *TERT* promoter variants in serial dilution samples containing 0.1%, 1%, 10%, and 30% variant‐positive DNA in a wild‐type background. The first column shows results for C228T and the second column for C250T. Within each column, the panels are arranged according to increasing expected variant allele frequency (VAF). Each dilution was analyzed in triplicate (*n* = 3 technical replicates per dilution), and representative plots are shown. The x‐axis represents VIC fluorescence intensity, and the y‐axis represents FAM fluorescence intensity.

The limit of detection (LOD) of the AbsoluteQ dPCR assays was evaluated using serial dilutions of DNA containing 0.1%, 1%, 10%, and 30% of either the C228T or C250T variant mixed with wild‐type DNA. The lowest variant allele frequency (VAF) reliably detected was 0.1% for both variants. To assess the quantitative linearity of allele detection, the experimentally determined VAFs were plotted against the expected VAFs based on the serial dilutions of input DNA (Fig. 2). The plots represent serial dilutions of a reference sample containing known variant fractions (0.1%, 1%, 10%, and 30%) each measured in triplicate. The observed VAF values are plotted against the expected VAF values. A linear regression line is shown in a Fig. 2 for the C228T and C250T variants, demonstrating a strong correlation (*R* ^2^ = 0.997) with a root mean square error (RMSE) of 0.580 for C228T. These results indicate high quantitative accuracy and reproducibility of the assays across a wide dynamic range. Also, for the C250T variant, a strong linear correlation was observed (*R* ^2^ = 0.992), with a root mean square error (RMSE) of 1.189. The low RMSE value of 0.580 indicates high concordance between expected and observed VAFs, demonstrating the assay's high accuracy and precision across the tested range. In contrast, an RMSE of 1.189 suggests greater variability, which may affect quantification accuracy, particularly at lower VAF levels based on our experience. The 10% dilution samples were responsible for the significantly higher RMSE values. Raw data generated by the AbsoluteQ Control Software are presented as scatter plots in Fig. 3.

### 3.3 Performance on clinical samples

**Fig. 4:**
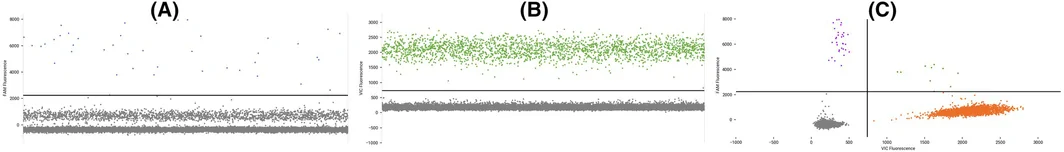
Representative result from the AbsoluteQ digital PCR (dPCR) assay in a urine sediment DNA sample positive for the *TERT* promoter C228T variant, with a measured variant allele frequency (VAF) of 1.71%. Panels show fluorescence signal distribution in the FAM channel (A), VIC channel (B), and the combined FAM/VIC plot (C). The figure shows one representative positive clinical sample (*n* = 1 sample shown) from the study cohort.

In this study, 113 samples were analyzed using the AbsoluteQ Digital PCR System, including 58 from the case group (BC) and 55 from the control group. A total of 47 samples were identified as positive for the C228T variant, and 5 samples for the C250T variant. Overall, 49 of 54 males (91%) and 3 of 4 females (75%) tested positive for either variant. Representative scatterplots obtained from digital PCR assays targeting *TERT* promoter variants in a positive clinical sample are shown in Fig. 4, including the FAM channel (Fig. 4A), VIC channel (Fig. 4B), and the combined FAM/VIC plot (Fig. 4C).

**Fig. 5:**
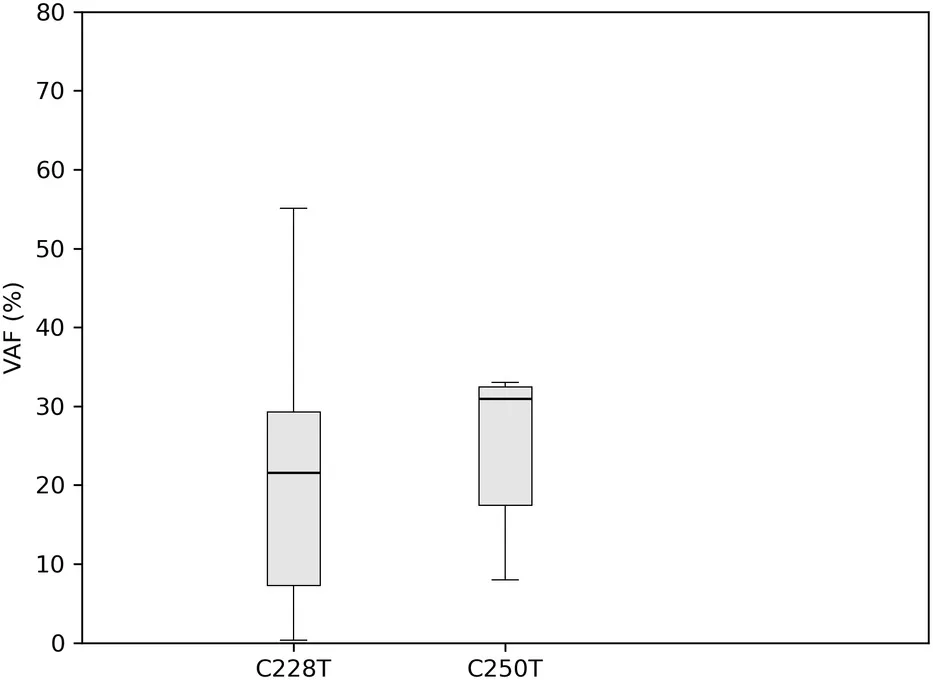
Distribution of variant allele frequency (VAF) values for the *TERT* promoter variants C228T (*n* = 47) and C250T (*n* = 5) detected in clinical samples. The central horizontal line indicates the median, the box indicates the interquartile range, and the whiskers indicate the range of observed values.

The variant allele frequency (VAF) distributions of the *TERT* promoter variants C228T and C250T demonstrated distinct patterns (Fig. 5). For the C228T variant (*n* = 47), VAFs ranged from 0.34% to 55.1%, with a mean of 20.06% (SD = 13.77). The distribution was broad, indicating substantial variability among samples, with 50% of cases (interquartile range: 7.26%–29.30%) showing moderate to high mutation burdens. In contrast, the C250T variant was detected in five samples, with a higher mean VAF of 24.35% (SD = 11.17), and a narrower range (8.01%–33.02%).

### 3.4 Diagnostic accuracy of sanger sequencing and AbsoluteQ Digital PCR

The diagnostic performance of three analytical methods for detecting TERT promoter variants was assessed based on a total of 113 samples: (1) AbsoluteQ Digital PCR, (2) Sanger sequencing with a confirmed variant on a single DNA strand (either forward or reverse), and (3) Sanger sequencing with variant confirmation on both DNA strands, representing the current diagnostic standard (Table 2). AbsoluteQ Digital PCR demonstrated the highest diagnostic accuracy, identifying 52 true positives and 55 true negatives, with no false positives and only 6 false negatives. This corresponded to a sensitivity of 89.65% (95% CI: 78.16–95.72) and a specificity of 100% (95% CI: 91.87–100). Sanger sequencing of a single DNA strand (either forward or reverse) showed reduced sensitivity, correctly detecting 40 true positives and 18 false negatives, resulting in a sensitivity of 68.97% (95% CI: 55.31–80.10). The lowest diagnostic performance was observed for Sanger sequencing of both DNA strands simultaneously (the current diagnostic standard) with a sensitivity of 50.00% (95% CI: 36.73–63.27), based on 29 true positives and 29 false negatives. In evaluating the analytical performance of the methods, AbsoluteQ Digital PCR demonstrated the highest accuracy (94.69%), outperforming Sanger sequencing interpreted using either forward or reverse reads (84.07%) as well as combined forward and reverse reads (74.34%).

**Table 2:** Diagnostic performance of AbsoluteQ Digital PCR and Sanger sequencing (1 strand or 2 strands).

	AbsoluteQ dPCR	Sanger F v. R	Sanger F + R
Total number analyzed, no	113	113	113
True positives, no	52	40	29
True negative, no	55	55	55
False positives, no	0	0	0
False negatives, no	6	18	29
Sensitivity (%, 95% CI)	89.65 (78.16–95.72)	68.97 (55.31–80.10)	50 (36.73–63.27)
Specificity (%, 95% CI)	100 (91.87–100)	100 (91.87–100)	100 (91.87–100)
Accuracy (%)	94.69	84.07	74.34

## 4 Discussion

*TERT* promoter variants C228T (−124 bp) and C250T (−146 bp) are among the earliest and most frequent genetic events in urothelial carcinogenesis and are readily shed into urine, making them compelling candidates for non‐invasive detection and surveillance of bladder cancer [24, 31]. In prospective population data, urinary *TERT* variants can be detected years before a clinical diagnosis, underscoring their value for early detection [32]. Multiple methodologies have been developed for the detection of *TERT* promoter variants, each with distinct advantages and limitations.

This study is the first to validate the use of AbsoluteQ Digital PCR for detecting *TERT* promoter variants in urinary sediment samples for the diagnosis of urothelial bladder cancer and to assess its diagnostic performance in comparison with a Sanger‐based approach. Sanger sequencing was selected as the reference method due to its widespread use in clinical laboratories, despite limited sensitivity at low variant allele frequencies.

Across platforms, reported diagnostic performance varies with assay, pre‐analytical handling, disease spectrum, and various types of biological material (cell‐free DNA from urine supernatant vs. cellular DNA from urine sediment). Sensitive next‐generation sequencing (NGS) and digital PCR assays consistently outperform earlier SNaPshot or Sanger‐based approaches in urine and are currently emerging as a new standard for the detection of *TERT* promoter variants in molecular diagnostics [28, 32, 33, 34]. The applicability of Sanger sequencing for *TERT* promoter variants detection is largely limited to tissue specimens, where the proportion of mutant DNA is relatively high, in contrast to urine samples, which typically contain a lower fraction of mutated alleles. In several studies, droplet digital PCR (ddPCR) showed greater sensitivity in detecting *TERT* promoter variants compared to conventional Sanger sequencing across various sample types, particularly in low VAF such as urine and FFPE tissue with reported sensitivity in urothelial carcinoma reaching ~89% for sensitive assays (e.g., castPCR) compared to ~50% for Sanger sequencing [34, 35, 36]. In an independent study conducted in two cohorts (France and Portugal), the UroMuTERT assay detected *TERT* variants with sensitivities of 87.1% (95% CI 78.6–93.2%) and 68.0% (95% CI 53.3–80.5) and specificities of 94.7% (95% CI 88.0–98.3%) and 98.0% (95% CI 89.3–100.0), respectively; combining urinary cfDNA and cellular DNA maximized detection and markedly outperformed cytology, particularly in low‐grade disease [33]. In another study, droplet digital PCR assays for *TERT* promoter variants (C228T/C250T) demonstrated a sensitivity of 86.8% (95% CI: 80.3–94.5) and a specificity of 92.4% (95% CI: 85.0–96.9) [37]. AbsoluteQ Digital PCR represents a digital PCR technology that enables rapid and highly precise absolute quantification of nucleic acids with minimal hands‐on time. In comparison to other digital PCR platforms, AbsoluteQ offers a simplified workflow, reduced turnaround time, and integrated sample partitioning and detection, while maintaining high analytical sensitivity and specificity. These features make it particularly well suited for the detection of low‐frequency variants in clinical samples, where robust performance and operational efficiency are critical.

Our results highlight that AbsoluteQ Digital PCR offers superior diagnostic performance compared to conventional Sanger sequencing for the detection of *TERT* promoter variants. Specifically, AbsoluteQ dPCR achieved a sensitivity of 89.65% (95% CI: 78.16–95.72) and a specificity of 100% (95% CI: 91.87–100), with no false positives. Our results are consistent with those reported in previous studies utilizing digital PCR for the detection of *TERT* promoter variants in urinary DNA. Therefore, the diagnostic performance observed in our Polish cohort is consistent with results from other populations, further supporting *TERT* promoter variants as a universal biomarker. In addition, our findings underscore the superior sensitivity of digital PCR, particularly for the detection of low‐frequency *TERT* promoter variants, and highlight its potential as a more effective tool for clinical diagnostics. Furthermore, our findings demonstrate that the applied digital PCR assay is capable of detecting *TERT* promoter variants at VAF as low as 0.1%, aligning with or exceeding the sensitivity thresholds reported in previous studies. Most ddPCR‐based assays for *TERT* variants have reported limits of detection in the range of 0.1%–0.2%, depending on assay conditions, input DNA quantity, and background noise levels [37, 38]. The ability to detect low‐frequency variants is particularly important in urine‐based testing, where tumor‐derived DNA is often highly diluted.

The overall sensitivity and specificity achieved by our single‐target *TERT* promoter dPCR assay compare favorably with alternative urine‐based approaches reported for primary detection of bladder cancer. Several urine assays have been evaluated in this setting. For example, in suspected urothelial carcinoma cohorts, Xpert® Bladder Cancer Detection showed sensitivities of approximately 90–96% with specificities of approximately 80–85% [39]. Similarly, a multigene urine RNA test (CX BLADDER) achieved 82% sensitivity at 85% specificity in a prospective gross‐hematuria cohort and was reported to outperform NMP22 and cytology in that setting [40]. A combined DNA methylation + mutation model (including *FGFR3*/*TERT*/*HRAS* variant analysis and methylation markers) reported 93% sensitivity and 86% specificity [41]. Other approaches include protein‐based assays such as ADXBLADDER (MCM5), which showed more moderate sensitivity with variable specificity across studies (e.g., 60.5% sensitivity and 88.2% specificity) in a diagnostic cohort [42]. In our cohort, our single‐target *TERT* promoter dPCR assay achieved very high specificity and sensitivity in the range of several published urine‐based assays. However, cross‐study comparisons should be interpreted with caution, as reported performance depends strongly on study design, cohort composition, reference standards, and tumor spectrum.

This study shares several limitations commonly reported in the *TERT* biomarker literature. The case–control design may lead to overestimation of specificity compared to prospective cohorts, highlighting the necessity for validation studies to accurately determine the true negative and positive predictive values. Despite efforts to assemble representative case and control groups, the case cohort showed a male overrepresentation most likely reflecting the markedly higher incidence of bladder cancer in men, whereas the control group was significantly enriched for women, which we attribute to greater willingness of healthy women to participate in the study and a relatively high rate of non‐NHGUC cytology results among apparently healthy men. The study design did not allow for systematic collection of complete clinical metadata for all participants, including the exact time interval between bladder cancer diagnosis and urine sample collection in the case group, and the precise histopathological subtypes or smoking status. Therefore, these variables were not analyzed in relation to the prevalence of *TERT* promoter variants. This limitation may have introduced spectrum bias and could have affected the estimated diagnostic performance if certain clinical subgroups were overrepresented in the available dataset. The substantial mean age difference of 19 years between the case and control groups, resulting from the study design, may have artificially increased the estimated assay specificity by underrepresenting age‐associated *TERT* promoter mutations that can be present in histologically normal urothelium [43]. Although these imbalances are not expected to significantly affect the analytical performance of the presented method, a residual impact cannot be definitively excluded. Despite optimized pre‐analytical conditions, urine remains a complex biological material, with factors such as processing time, storage, inflammation, and recent instrumentation influencing mutant DNA concentration. Like other targeted assays, this approach focuses on canonical hotspot mutations, potentially missing non‐canonical or structural alterations without complementary sequencing panels [37].

In the future integrative diagnostic approaches combining *TERT* promoter analysis with complementary biomarkers such as *FGFR3* variants, DNA methylation signatures, or cytology have the potential to improve overall sensitivity, while carefully balancing specificity and maintaining clinical feasibility [44]. Furthermore, longitudinal monitoring of variant allele fractions before and after tumor resection may enable risk‐adapted surveillance strategies and earlier intervention, supported by evidence that *TERT* positivity can precede clinical detection by several years.

## 5 Conclusion

Our study provides clinically relevant evidence that a streamlined, single‐gene *TERT* promoter assay can be implemented with robust analytical performance and operational efficiency. Using an optimized digital PCR protocol on urinary DNA, we achieved high discrimination between cases and controls and reliably detected low‐frequency variants, consistent with prior findings on the advantages of digital quantification for GC‐rich *TERT* sequence.

In summary, our results highlight the superior analytical performance of the AbsoluteQ Digital PCR compared to Sanger sequencing and support its potential integration into clinical workflows for the sensitive and reliable detection of *TERT* promoter mutations in bladder cancer, particularly in samples with ultra‐low VAF.

## Conflict of interest

The authors declare no conflicts of interests.

## Author contributions

TK conceived and designed the study and contributed to writing the manuscript. AN developed and established the method, performed the dPCR experiments, and contributed to writing the manuscript. IK and ŻK performed DNA isolation and participated in data acquisition. LR performed the dPCR experiments and participated in data acquisition. ŁK conducted the statistical analysis. MB participated in data acquisition and study logistics. DS, JW, PK, and AJ participated in data acquisition. BK and AG supervised the study and critically revised the manuscript. All authors read and approved the final manuscript.

## Data Availability

The data and materials used in this study are available from the corresponding author upon reasonable request.

## References

[1] Bray F , Laversanne M , Sung H , Ferlay J , Siegel RL , Soerjomataram I , et al. Global cancer statistics 2022: GLOBOCAN estimates of incidence and mortality worldwide for 36 cancers in 185 countries. CA Cancer J Clin. 2024;74:229–263. 10.3322/caac.21834 38572751

[2] Didkowska J , Barańska K , Miklewska MJ , Wojciechowska U . Cancer incidence and mortality in Poland in 2023. Nowotwory Journal of Oncology. 2024;74:75–93. 10.5603/njo.99065

[3] Bracarda S , Iacovelli R , Baldazzi V , Zucali PA , Gernone A , Conti GN , et al. U‐CHANGE project: a multidimensional consensus on how clinicians, patients and caregivers May approach together the new urothelial cancer scenario. Front Oncol. 2023;13:1186103. 10.3389/fonc.2023.1186103 37576880 PMC10422043

[4] Sievert KD , Amend B , Nagele U , Schilling D , Bedke J , Horstmann M , et al. Economic aspects of bladder cancer: what are the benefits and costs? World J Urol. 2009;27:295–300. 10.1007/s00345-009-0395-z 19271220 PMC2694315

[5] Jocham D , Stepp H , Waidelich R . Photodynamic diagnosis in urology: state‐of‐the‐art. Eur Urol. 2008;53:1138–1148. 10.1016/j.eururo.2007.11.048 18096307

[6] Lee H‐H , Kim SH . Review of non‐invasive urinary biomarkers in bladder cancer. Transl Cancer Res. 2020;9:6554–6564. 10.21037/tcr-20-1990 35117265 PMC8798424

[7] Leiblich A . Recent developments in the search for urinary biomarkers in bladder cancer. Curr Urol Rep. 2017;18:100. 10.1007/s11934-017-0748-x 29130146 PMC5682315

[8] Yafi FA , Brimo F , Steinberg J , Aprikian AG , Tanguay S , Kassouf W . Prospective analysis of sensitivity and specificity of urinary cytology and other urinary biomarkers for bladder cancer. Urol Oncol. 2015;33:66.e25‐31. 10.1016/j.urolonc.2014.06.008 25037483

[9] Burchardt M , Burchardt T , Shabsigh A , De La Taille A , Benson MC , Sawczuk I . Current concepts in biomarker Technology for Bladder Cancers. Clin Chem. 2000;46:595–605. 10794739

[10] Lotan Y , Roehrborn CG . Sensitivity and specificity of commonly available bladder tumor markers versus cytology: results of a comprehensive literature review and meta‐analyses. Urology. 2003;61:109–118; discussion 118. 10.1016/s0090-4295(02)02136-2 12559279

[11] Grossman HB , Messing E , Soloway M , Tomera K , Katz G , Berger Y , et al. Detection of bladder cancer using a point‐of‐care proteomic assay. JAMA. 2005;293:810–816. 10.1001/jama.293.7.810 15713770

[12] Roobol MJ , Bangma CH , el Bouazzaoui S , Franken‐Raab CG , Zwarthoff EC . Feasibility study of screening for bladder cancer with urinary molecular markers (the BLU‐P project). Urol Oncol. 2010;28:686–690. 10.1016/j.urolonc.2009.12.002 21062653

[13] Starke N , Singla N , Haddad A , Lotan Y . Long‐term outcomes in a high‐risk bladder cancer screening cohort. BJU Int. 2016;117:611–617. 10.1111/bju.13154 25891519

[14] Sharma G , Sharma A , Krishna M , Devana SK , Singh SK . Xpert bladder cancer Monitor in surveillance of bladder cancer: systematic review and meta‐analysis. Urol Oncol. 2022;40:e1–e9. 10.1016/j.urolonc.2021.08.017 34535354

[15] Fiorentino V , Pizzimenti C , Franchina M , Rossi ED , Tralongo P , Carlino A , et al. Bladder Epicheck test: a novel tool to support urothelial carcinoma diagnosis in urine samples. Int J Mol Sci. 2023;24:12489. 10.3390/ijms241512489 37569864 PMC10420163

[16] Kavalieris L , O'Sullivan P , Frampton C , Guilford P , Darling D , Jacobson E , et al. Performance characteristics of a multigene urine biomarker test for monitoring for recurrent urothelial carcinoma in a multicenter study. J Urol. 2017;197:1419–1426. 10.1016/j.juro.2016.12.010 27986532

[17] Kravchuk AP , Wolff I , Gilfrich C , Wirtz RM , Soares P , Braun K‐P , et al. Urine‐based biomarker test Uromonitor® in the detection and disease monitoring of non‐muscle‐invasive bladder cancer‐a systematic review and meta‐analysis of diagnostic test performance. Cancers (Basel). 2024;16:753. 10.3390/cancers16040753 38398144 PMC10886463

[18] Ward DG , Baxter L , Ott S , Gordon NS , Wang J , Patel P , et al. Highly sensitive and specific detection of bladder cancer via targeted ultra‐deep sequencing of urinary DNA. Eur Urol Oncol. 2023;6:67–75. 10.1016/j.euo.2022.03.005 35410825

[19] Bonetti D , Martina M , Falcettoni M , Longhese MP . Telomere‐end processing: mechanisms and regulation. Chromosoma. 2013;123:57–66. 10.1007/s00412-013-0440-y 24122006

[20] Muraki K , Nyhan K , Han L , Murnane JP . Mechanisms of telomere loss and their consequences for chromosome instability. Front Oncol. 2012;2:135. 10.3389/fonc.2012.00135 23061048 PMC3463808

[21] Shay JW , Bacchetti S . A survey of telomerase activity in human cancer. Eur J Cancer. 1997;33:787–791. 10.1016/S0959-8049(97)00062-2 9282118

[22] Kim NW , Piatyszek MA , Prowse KR , Harley CB , West MD , Ho PL , et al. Specific Association of Human Telomerase Activity with immortal cells and cancer. Science. 1994;266:2011–2015. 10.1126/science.7605428 7605428

[23] Counter CM , Meyerson M , Eaton EN , Ellisen LW , Caddle SD , Haber DA , et al. Telomerase activity is restored in human cells by ectopic expression of hTERT (hEST2), the catalytic subunit of telomerase. Oncogene. 1998;16:1217–1222. 10.1038/sj.onc.1201882 9528864

[24] Allory Y , Beukers W , Sagrera A , Flández M , Marqués M , Márquez M , et al. Telomerase reverse transcriptase promoter mutations in bladder cancer: high frequency across stages, detection in urine, and lack of association with outcome. Eur Urol. 2014;65:360–366. 10.1016/j.eururo.2013.08.052 24018021

[25] Borah S , Xi L , Zaug AJ , Powell NM , Dancik GM , Cohen SB , et al. Cancer. TERT promoter mutations and telomerase reactivation in urothelial cancer. Science. 2015;347:1006–1010. 10.1126/science.1260200 25722414 PMC4640672

[26] Colebatch AJ , Dobrovic A , Cooper WA . TERT gene: its function and dysregulation in cancer. J Clin Pathol. 2019;72:281–284. 10.1136/jclinpath-2018-205653 30696697

[27] Mitas M , Yu A , Dill J , Kamp TJ , Chambers EJ , Haworth IS . Hairpin properties of single‐stranded DNA containing a GC‐rich triplet repeat: (CTG)15. Nucleic Acids Res. 1995;23:1050–1059. 10.1093/nar/23.6.1050 7731793 PMC306804

[28] Corless BC , Chang GA , Cooper S , Syeda MM , Shao Y , Osman I , et al. Development of novel mutation‐specific droplet digital PCR assays detecting TERT promoter mutations in tumor and plasma samples. J Mol Diagn. 2019;21:274–285. 10.1016/j.jmoldx.2018.09.003 30827467 PMC6419583

[29] da Costa VR , Bim LV , LD P e S , Colloza‐Gama GA , Bastos AU , Delcelo R , et al. Advances in detecting low prevalence somatic TERT promoter mutations in papillary thyroid carcinoma. Front Endocrinol (Lausanne). 2021;12:643151. 10.3389/fendo.2021.643151 33776938 PMC7994758

[30] Muralidharan K , Yekula A , Small JL , Rosh ZS , Kang KM , Wang L , et al. TERT promoter mutation analysis for blood‐based diagnosis and monitoring of gliomas. Clin Cancer Res. 2021;27:169–178. 10.1158/1078-0432.CCR-20-3083 33051308 PMC7785705

[31] Kinde I , Munari E , Faraj SF , Hruban RH , Schoenberg M , Bivalacqua T , et al. TERT promoter mutations occur early in urothelial neoplasia and are biomarkers of early disease and disease recurrence in urine. Cancer Res. 2013;73:7162–7167. 10.1158/0008-5472.CAN-13-2498 24121487 PMC3966102

[32] Hosen MI , Sheikh M , Zvereva M , Scelo G , Forey N , Durand G , et al. Urinary TERT promoter mutations are detectable up to 10 years prior to clinical diagnosis of bladder cancer: evidence from the Golestan cohort study. EBioMedicine. 2020;53:102643. 10.1016/j.ebiom.2020.102643 32081602 PMC7118568

[33] Avogbe PH , Manel A , Vian E , Durand G , Forey N , Voegele C , et al. Urinary TERT promoter mutations as non‐invasive biomarkers for the comprehensive detection of urothelial cancer. EBioMedicine. 2019;44:431–438. 10.1016/j.ebiom.2019.05.004 31122840 PMC6603852

[34] Adachi J‐I , Shirahata M , Suzuki T , Mishima K , Uchida E , Sasaki A , et al. Droplet digital PCR assay for detecting TERT promoter mutations in patients with glioma. Brain Tumor Pathol. 2021;38:201–209. 10.1007/s10014-021-00403-4 34128111

[35] Wang K , Liu T , Ge N , Liu L , Yuan X , Liu J , et al. TERT promoter mutations are associated with distant metastases in upper tract urothelial carcinomas and serve as urinary biomarkers detected by a sensitive castPCR. Oncotarget. 2014;5:12428–12439. 10.18632/oncotarget.2660 25474136 PMC4322995

[36] Otsuji K , Sasaki T , Tanabe M , Seto Y . Droplet‐digital PCR reveals frequent mutations in TERT promoter region in breast fibroadenomas and phyllodes Tumours, irrespective of the presence of MED12 mutations. Br J Cancer. 2021;124:466–473. 10.1038/s41416-020-01109-8 33046803 PMC7852881

[37] Hosen MI , Forey N , Durand G , Voegele C , Bilici S , Avogbe PH , et al. Development of sensitive droplet digital PCR assays for detecting urinary TERT promoter mutations as non‐invasive biomarkers for detection of urothelial cancer. Cancers (Basel). 2020;12:3541. 10.3390/cancers12123541 33260905 PMC7761513

[38] McEvoy AC , Calapre L , Pereira MR , Giardina T , Robinson C , Khattak MA , et al. Sensitive droplet digital PCR method for detection of TERT promoter mutations in cell free DNA from patients with metastatic melanoma. Oncotarget. 2017;8:78890–78900. 10.18632/oncotarget.20354 29108273 PMC5668006

[39] Kavcic N , Peric I , Zagorac A , Kokalj Vokac N . Clinical evaluation of two non‐invasive genetic tests for detection and monitoring of urothelial carcinoma: validation of UroVysion and Xpert bladder cancer detection test. Front Genet. 2022;13:839598. 10.3389/fgene.2022.839598 35734425 PMC9208547

[40] O'Sullivan P , Sharples K , Dalphin M , Davidson P , Gilling P , Cambridge L , et al. A multigene urine test for the detection and stratification of bladder cancer in patients presenting with hematuria. J Urol. 2012;188:741–747. 10.1016/j.juro.2012.05.003 22818138

[41] van Kessel KEM , Beukers W , Lurkin I , Ziel‐van der Made A , van der Keur KA , Boormans JL , et al. Validation of a DNA methylation‐mutation urine assay to select patients with hematuria for cystoscopy. J Urol. 2017;197:590–595. 10.1016/j.juro.2016.09.118 27746284

[42] Anastasi E , Maggi M , Tartaglione S , Angeloni A , Gennarini G , Leoncini PP , et al. Predictive value of MCM5 (ADXBLADDER) analysis in urine of men evaluated for the initial diagnosis of bladder cancer: a comparative prospective study. Diagn Cytopathol. 2020;48:1034–1040. 10.1002/dc.24530 32562513

[43] Calvet F , Blanco Martinez‐Illescas R , Muiños F , Tretiakova M , Latorre‐Esteves ES , Fredrickson J , et al. Sex and smoking bias in the selection of somatic mutations in human bladder. Nature. 2025;647:436–444. 10.1038/s41586-025-09521-x 41062697 PMC12611770

[44] Springer SU , Chen C‐H , Rodriguez Pena MDC , Li L , Douville C , Wang Y , et al. Non‐invasive detection of urothelial cancer through the analysis of driver gene mutations and aneuploidy. elife. 2018;7:e32143. 10.7554/eLife.32143 29557778 PMC5860864

